# Optimising the MR‐Linac as a standard treatment modality

**DOI:** 10.1002/jmrs.712

**Published:** 2023-08-04

**Authors:** Jeremy de Leon, Tania Twentyman, Madeline Carr, Michael Jameson, Vikneswary Batumalai

**Affiliations:** ^1^ GenesisCare Alexandria New South Wales Australia; ^2^ Centre for Medical Radiation Physics University of Wollongong Wollongong New South Wales Australia; ^3^ School of Clinical Medicine, Faculty of Medicine and Health UNSW Sydney Sydney New South Wales Australia

## Abstract

The magnetic resonance linear accelerator (MR‐Linac) offers a new treatment paradigm, providing improved visualisation of targets and organs at risk while allowing for daily adaptation of treatment plans in real time. Online MR‐guided adaptive treatment has reduced treatment uncertainties; however, the additional treatment time and resource requirements may be a concern. We present our experience of integrating an MR‐Linac into a busy department and provide recommendations for improved clinical and resource efficiency. Furthermore, we discuss potential future technological innovations that can further optimise clinical productivity in a busy department.

## Graphical abstract

The MR‐Linac introduces a novel approach to radiotherapy by combining improved visualisation capabilities with real‐time adaptation of treatment plans. While online MR‐guided adaptive treatment reduces uncertainties, it also raises concerns about increased treatment time and resource demands. This article shares insights from integrating an MR‐Linac into a busy department, offering recommendations for enhanced clinical and resource efficiency.

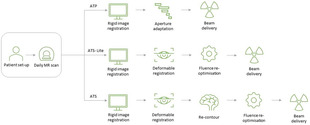

## Introduction

Online magnetic resonance (MR)‐guided radiation therapy involves using a hybrid MR linear accelerator (MR‐Linac) to deliver radiotherapy treatment. It provides enhanced soft‐tissue visualisation and real‐time motion monitoring during treatment. Additional benefits include the ability to adapt plans based on daily anatomy, potentially reducing margins and improving accuracy. Early evidence indicates good patient tolerance with acceptable toxicity profile.^1^, ^2^ The Elekta Unity MR‐Linac system (Elekta AB, Stockholm, Sweden) is one of the main clinically operational platforms and the focus of this article.

We previously reported our initial 6 months of clinical operations highlighting the training requirements and nuances of treating using this technology.^3^ In this article, we share our experience and recommendations for integrating an MR‐Linac into a busy department, as well as processes to enhance clinical and resource efficiency. In addition, we explore future technological advancements that may further improve clinical efficiency.

## Clinical Experience

**Figure 1:**
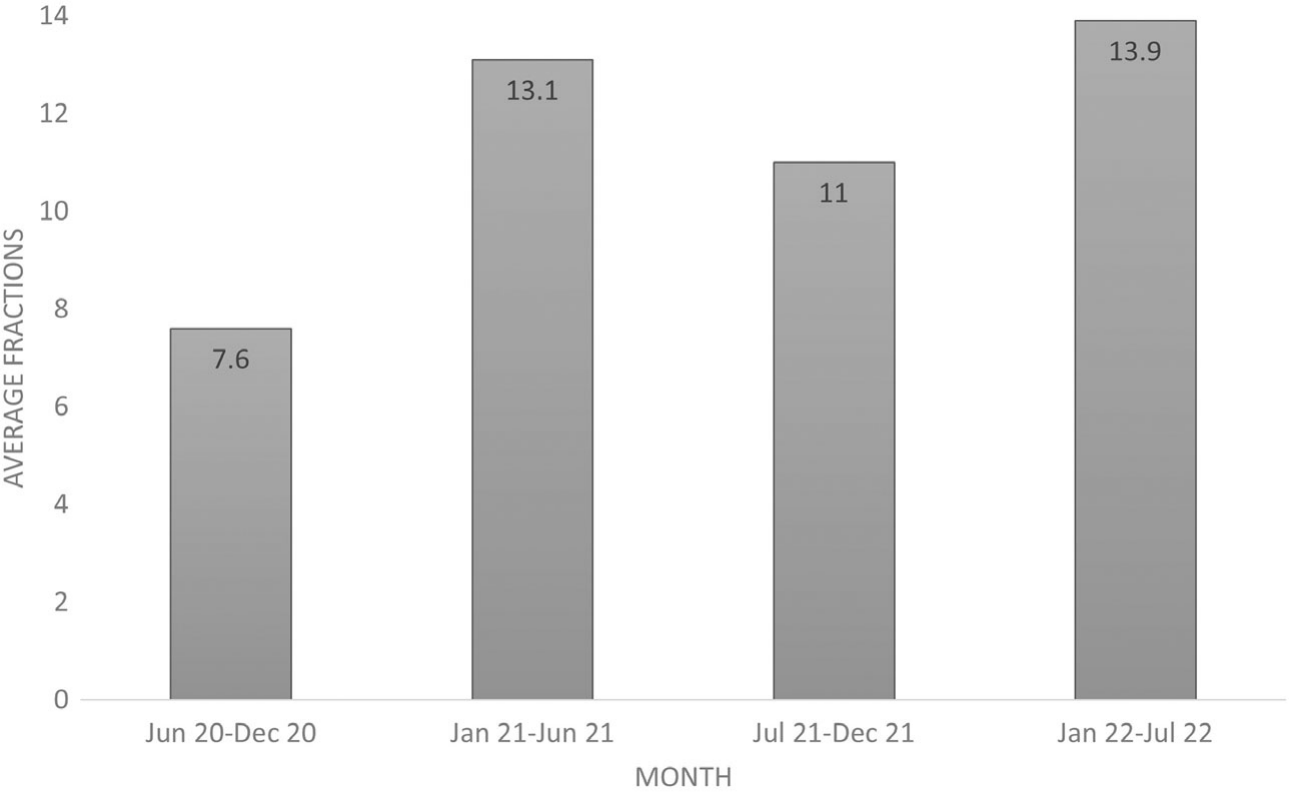
Average number of fractions per day for the various time periods.

The MR‐Linac program at St Vincent's Sydney in GenesisCare Australia launched in July 2020, and while the initial clinical ramp‐up was gradual, significant improvements in treatment efficiency were achieved over the first year. For stereotactic treatments (dose per fraction 5–14 Gy), average treatment times reduced from 57 min in the first 6 months to 42 min in the next 6 months. Similarly, for long‐course treatments (>15 fractions), average treatment times reduced from 42 min in the first 6 months to 35 min in the next 6 months. These improvements have been sustained over time, despite the increasing treatment complexity, for example stereotactic body radiotherapy (SBRT) and new clinical indications. These efficiencies can be attributed to several factors including the increased confidence and experience of the dedicated multidisciplinary team, improvements in documentation and communication between the treating team and the implementation of a Wacom Cintiq tablet (Wacom Co., Ltd., Otone, Saitama, Japan) to reduce online contouring time.^4^ Ongoing staff education and training are crucial for maintaining efficiency gains.^5^ In the first 2 years, the most frequent treatment sites were prostate (40%), oligometastatic lymph node (30%), liver (8%) and pancreas (8%). Treatment capacity increased significantly over time, with the average number of fractions per day increasing from 7.6 to 13.9 between June–December 2020 and January–July 2022 (Fig. 1).

The evidence supporting the benefits of MR‐guided adaptive radiotherapy (MRgART) compared with conventional radiotherapy is not yet mature.^6^ To aid in gathering adequate evidence, we developed the ADAPT‐MRL (Analysis of data to Advance Personalised Therapy with MR‐Linac) registry.^7^ The registry aims to collect both clinical and research data for patients treated on the MR‐Linac. Early results for localised prostate cancer patients show that ultra‐hypofractionated MR‐Linac treatment is safe and effective, with no grade ≥3 toxicity reported.^8^ The registry is an ongoing initiative that will continue to collect data and evidence on patient outcomes and toxicity for all MR‐Linac patients to assess long‐term clinical outcomes.

## Efficiency in Treatment Workflow

**Figure 2:**
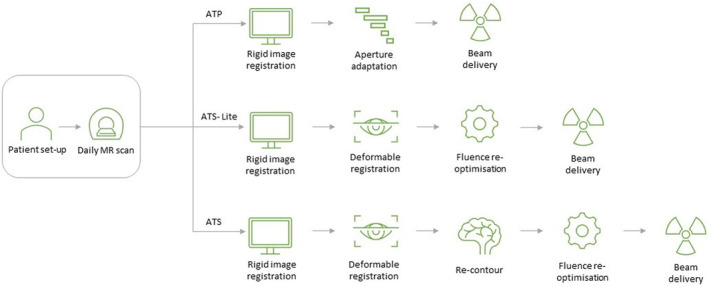
MR‐Linac workflow. ATP, adapt‐to‐position; ATS, adapt‐to‐shape.

The Unity system provides two modes for online adaptive treatments: adapt‐to‐position (ATP) and adapt‐to‐shape (ATS) (Fig. 2). The ATS workflow is a fully adaptive process that involves recontouring and replanning while the patient remains on the treatment couch. Our daily adaptive (replanning) strategy is designed around two key approaches: restoring the initial treatment intent and isotoxic dose escalation. For cases where the treatment intent is restored, plans are reoptimised based on the anatomy of the day using the same optimisation criteria and goals as the reference plan. Isotoxic dose escalation involves maximising the dose to the target volume while ensuring that organ at risk (OAR) tolerances are respected. In the early stages of operation, all patients were treated with an ATS workflow. While the ATS workflow is the most robust solution to adaptive radiotherapy,^9^ it is also the most resource‐intensive workflow. With increasing experience, we actively sought ways to improve efficiency while maintaining the benefits of daily adaptive treatment. This led us to adopt ATP or adapt‐to‐shape‐lite (ATS‐Lite) workflows for specific clinical scenarios based on the observed daily anatomy.

The ATP workflow involves a virtual isocentre shift and does not require recontouring. This approach is employed when a patient's anatomy is favourable and closely matches prior imaging. In our practice, for treatment involving more than five fractions, the first three to five fractions are treated with an ATS approach, and the resulting plans are used to create a plan library for the patient. Subsequent fractions are matched to the reference plan or an appropriate previous fraction. This approach requires manual selection of the most suitable plan and provides multiple options with different anatomical positions for that patient. This approach can speed up optimisation and shorten treatment times by eliminating the need for recontouring and is particularly useful in cases with stable anatomy of both target and OARs, such as oligometastatic lymph nodes.

The ATS‐Lite workflow is a useful approach in situations where the target is stable and matches well to the reference scan or previous fractions, but the patient contours or nearby OARs vary in position. This is a modification of the ATS workflow, where only one or two contours are edited. If the change is not large, the system's deformable image registration algorithm can often accommodate these changes automatically and quickly, eliminating the need for manual recontouring. This reduces contouring time while still utilising the ability to accurately adapt to anatomical changes and optimise the plan. We have successfully used this approach in stereotactic liver treatments and head and neck cases. Other reports have also shown similar use of the ATS‐Lite workflow for head and neck cases.^10^

During the initial 2 years of treatment, our clinic adapted 94.4% of fractions using the ATS or ATS‐Lite workflow, with the remaining fractions adapted using the ATP workflow. The MR‐Linac system's flexibility enables us to interchangeably use the appropriate workflow based on the daily anatomy.

## Reduced Resource Requirements

Due to the adaptive nature of MR‐Linac treatments, the workflow in most centres requires the presence of the RO, especially during recontouring. To address this, we have developed a training and credentialing process that enables radiation therapists to contour patient anatomy, similar to practices implemented in other centres.^11^, ^12^ Recent reports also show radiation therapists undertaking plan review based on predefined criteria.^12^ These solutions can help streamline the MR‐Linac treatment process and make it more similar to standard linac processes. Other solutions include simulation‐free treatment and artificial intelligence (AI)‐based contouring.

### Simulation‐free treatment

The MR‐Linac has the potential to remove the need for simulation and reference imaging for radiotherapy planning. While previous trials investigating simulation‐free treatment have mainly focused on palliative scenarios using conformal treatment techniques,^13^ the adaptive capabilities of the MR‐Linac may allow for more complex treatments with the use of diagnostic images as a ‘pseudo‐CT’. We are currently investigating the feasibility of using prostate‐specific membrane antigen positron emission tomography/CT (PSMA‐PET/CT) in lieu of simulation CT in an MR‐Linac workflow for definitive male pelvis treatment.^14^ Moving beyond pseudo‐CT and removing the need for any CT for planning can be achieved through MR‐only planning. However, MR‐only planning is limited by the lack of electron density distribution required for dose calculation. To overcome this limitation, synthetic CT or pseudo‐CT from MR images for treatment planning has been investigated.^15^, ^16^ More recently, commercially available solutions such as MRiPlanner (Spectronic Medical AB, Helsingborg, Sweden), MR‐Box™ (Therapanacea, Paris, France) and MRCAT (Philips Healthcare) is providing MR‐only workflow where the synthetic CT replaces simulation CT throughout all steps in the radiotherapy workflow. A future goal would be to adopt these approaches to allow rapid treatment workflow including same‐day treatment.

### AI‐based contouring

The delineation of target and OARs remains the most laborious and time‐consuming task and is a hindrance to adaptive radiotherapy in a busy department. Auto‐contouring methods have progressed over the last decade, with the most recent techniques involving AI approaches. Ongoing validation of AI‐based auto‐contouring tools has showed high contouring accuracy with less time and able to supplement clinicians with a quick solution.^17^, ^18^ This solution is an essential step towards the application of a rapid workflow in MRgART. This will not only improve treatment time but also monitor anatomical changes that may occur during the adaptive planning phase. Several commercially available AI‐based auto‐contouring tools for MR images have emerged in recent years including Contour ProtegeAI (MIM Software Inc., Cleveland, OH),^19^ Annotate ART‐Plan (Therapanacea, Paris, France)^20^ and Philips automated segmentation tool (Philips RTdrive Core 2.0, Philips Healthcare).^21^

## Advanced Treatment Options

Other important technological innovations, which could be achieved with MR‐Linac, include volumetric modulated arc therapy (VMAT) and multi‐leaf collimator (MLC) tracking, dose accumulation and imaging biomarkers.

### Volumetric modulated arc therapy and multi‐leaf collimator tracking

While the unique characteristics and design of the Unity MR‐Linac offer many advantages, there are some treatment delivery improvements that can be made. The Unity MR‐Linac offers a 7 MV flattening‐filter free (FFF) beam to be used for static intensity‐modulated radiotherapy (IMRT). On a standard linac, VMAT reduces delivery times when combined with high‐dose‐rate FFF beams but currently not available on the MR‐Linac system for clinical use. A recent proof‐of‐concept study showed that VMAT is feasible on the Unity system for prostate SBRT.^22^ However, the use of IMRT and VMAT increases the need for motion management, which can be a challenge. While gating is the solution to manage motion, this significantly increases treatment time.^23^ An alternative solution is MLC tracking, which realigns the treatment beam with the most recently observed (or predicted) tumour position.^24^ A recent experimental demonstration of VMAT combined with MLC tracking for lung SBRT showed that it is technically feasible on the MR‐Linac and results in highly conformal dose distribution.^25^

### Dose accumulation

While the daily plan adaptation with MRgART offers personalised treatment approach, the accuracy of the assessment of the daily delivered dose would further improve through dose accumulation. If clinicians are able to review the deposited dose before each fraction, the plan of the day could be further personalised and optimised. Early studies showed that beam‐on MR‐Linac can be used for time‐resolved dose accumulation.^26^ More recently, it has been shown that dose accumulation strategy was able to measure accumulated dose to OAR and predict acute toxicities.^27^

### Imaging biomarkers

Quantitative imaging biomarkers (QIBs) can provide measurable characteristics about the physiology of a tissue being imaged (e.g. tissue cell density). MR imaging‐derived QIBs are of particular interest in oncology due to the vast literature suggesting they can assist in predicting treatment outcomes and assessing treatment responses.^28^, ^29^ Due to the infeasibility of using diagnostic MR systems for frequent QIB measurements, the literature is currently lacking large‐scale patient datasets to validate the clinical utility of QIBs.^30^ A hybrid MR‐Linac system provides the unique opportunity to acquire daily physiological images of a patient simultaneously to their treatment. This significantly reduces patient burden and department resources, and thus has potential to provide the large‐scale datasets required for QIB clinical validation.^31^ Furthermore, QIBs derived from MR‐Linacs introduce the prospective of using biological image‐guided adaptive radiotherapy, for example changing the dose distribution based on observed physiological tissue changes over the course of treatment.^32^

## Recommendations

The MR‐Linac's versatility in treating various anatomical sites makes it suitable for routine clinical use, unlike specialised machines such as CyberKnife and Gamma Knife. Based on our experience, new MR‐Linac centres should prioritise commonly treated anatomical sites. This includes sites where the soft‐tissue contrast using MRgART will allow safe high‐precision treatment with optimal sparing of healthy tissues, such as localised prostate cancer. Moving targets including pancreatic cancer, liver cancer and pelvic lymph nodes close to sensitive OARs will also benefit from MRgART with motion management approaches. These recommendations align with ESTRO's patient selection guidelines for MR‐Linac.^33^

The integration of an MR‐Linac into routine practice necessitates specific training and credentialing of staff to ensure safe and effective utilisation of the technology. We have developed a robust training and credentialing model encompassing essential areas including MR safety, MR image acquisition and optimisation for radiotherapy purposes, image interpretation and adaptive radiotherapy strategies.^5^ The program is designed to upskill staff members in a systematic and timely manner and includes annual refreshers to ensure that staff members maintain proficiency and stay up to date with the evolving best practices. The Australasian College of Physical Scientists and Engineers in Medicine (ACPSEM) has also recently published a position paper addressing the safety of MR‐Linacs, including recommendations on staff training levels and responsibilities.^34^

Our staffing model for the MR‐Linac is comparable to a standard linac, with a similar number of radiation therapists and radiation oncology medical physicists (ROMP) assigned per machine. This approach aligns with the position statement on MR‐Linac staffing model by the Australian Society of Medical Imaging and Radiation Therapy (ASMIRT).^35^ According to ASMIRT's recommendations, the MR‐Linac should have a staffing configuration similar to standard linacs, with six to eight fully trained full‐time radiation therapists.^35^ In addition to the traditional core team consisting of radiation therapists, ROMPs and ROs, the integration of MR radiographers in the staffing model may also be an important requirement.^36^ Considering the extensive education and training requirements, we suggest establishing a core team for the MR‐Linac, minimising rotations to other treatment areas during the initial period. This will not only increase proficiency in the core team and ensure the retention of trained and upskilled staff, but also maintain MR safety and mitigate the risk of any potential catastrophic accidents. This approach could aid a department in strategizing their long‐term goals for acquiring an MR‐Linac, thus ensuring the sustainability of their MR‐Linac program. Currently, we employ an ‘RO of the day’ approach, dedicating one RO to MR‐Linac treatment each day, resulting in a streamlined treatment delivery process. Furthermore, transitioning to radiation therapist‐led treatment^37^ and utilising a combination of ATP, ATS and ATS‐Lite workflows will reduce the need for ROs at the console, aligning resource requirements with those of a standard linac treatment.

Treatment interruptions on the MR‐Linac are rare in our experience; therefore, dual planning (creating backup plans for standard linac) is unnecessary. In 2 years of operation, we encountered only one significant MR‐Linac breakdown, necessitating the creation of new plans for patients to transition to a standard linac. Additionally, our high volume of SBRT cases enables longer breaks between fractions compared with extended treatments.

## Challenges

Despite superior image quality, MR‐Linac systems can still face limitations in visualising tumours affected by breathing artefact, particularly in the upper and mid abdomen. Abdominal compression is recommended to help reduce the breathing artefacts. Additionally, large tumours may be difficult to treat due to the limited field of view (22 cm) in the superior–inferior direction. Patient intolerance and MR safety also need to be considered. Although patients with cardiovascular implantable electronic devices can be treated,^38^ lack of required MR‐compatible monitoring equipment prevents us from doing so as a departmental policy. This policy affected fewer than 10 patients in the first 2 years of operation.

While adaptive radiotherapy offers a promising solution to high‐precision treatment, there are also some important limitations that should be acknowledged. Long‐term outcomes of MRgART remain uncertain. Furthermore, tumours frequently respond to radiotherapy and thus become smaller over the course of treatment; however, it is unclear if it is safe to adapt to these smaller volumes as the region originally occupied by the tumour might still contain microscopic disease.^39^ This may lead to underdosage of microscopic disease, potentially increasing the risk of tumour recurrence. Accurately and prospectively collecting outcomes of patients treated on MR‐Linacs through initiatives such as the ADAPT‐MRL registry and the MOMENTUM study^1^ will aid in answering these unknowns about MRgART.

## Conclusions

MR‐Linac offers an advanced form of radiotherapy delivery with the potential to reduce treatment toxicity and improve clinical outcomes. The system has introduced different adaptive workflows and processes which may be seen as a barrier to general implementation and use of the technology. However, workflow efficiencies can be achieved through many solutions including treatment workflow flexibility, simulation‐free treatment, AI solutions and technological innovations. Upskilling of staff is required, however, as with any new technology, experience and improved communication can facilitate its routine use. Moreover, aside from clinical efficiency, the MR‐Linac presents opportunities for greater personalisation of treatment that could potentially revolutionise radiotherapy treatments in the future.

## Conflict of interest

The authors declare no conflict of interest.

## Data Availability

Data sharing not applicable to this article as no datasets were generated or analysed during the current study.
